# Changing Language May Not Be Enough to Change Public Perceptions of Individuals Who Sexually Offend

**DOI:** 10.5964/sotrap.17345

**Published:** 2026-05-08

**Authors:** Chella M. Robles, Harleen Cheema, Karen Buro, Sandy Jung

**Affiliations:** 1 Department of Psychology, MacEwan University, Edmonton, Alberta, Canada; 2 Department of Mathematics and Statistics, MacEwan University, Edmonton, Alberta, Canada; University of Lincoln, Lincoln, United Kingdom

**Keywords:** sexual offending, individuals who commit sexual offences, labels, stigma, person-first language

## Abstract

Negative community attitudes toward individuals who have sexually offended may hinder their reintegration, potentially leading to reoffending. The ‘sex offender’ label is believed to reinforce these negative perceptions. This study examines public perceptions of these individuals using person-first language compared to stigmatizing labels. Participants read one of eight public announcement vignettes and answered questions regarding their perceptions of individuals who sexually offend. Contrary to expectations, we found that participants continued to endorse negative perceptions toward these individuals irrespective of the label used, the victim’s age, or the interaction of the label and the victim’s age, suggesting that the labels were not perceived differently. In light of these null findings, adopting person-first language may not be enough to change societal attitudes toward individuals who sexually offend. Changing language should be considered when the goal is to treat individuals more humanely and to better align with trauma-informed and compassionate approaches when working with those who have been convicted of sexual offences.

## Abstract (plain language summary)

**Background:**

Individuals who have committed sexual offences are often portrayed in the media as high-risk, predatory, and incurable. These portrayals can foster negative community attitudes, which may hinder their successful reintegration. Poor community reintegration is concerning, as it is linked to factors that increase the likelihood of reoffending among individuals convicted of sexual offences. Research has shown that the ‘sex offender’ label reinforces these negative attitudes, perpetuating harmful stereotypes, such as the belief that all individuals who commit sexual offenses are inherently high-risk, dangerous, and incapable of change.

In contrast, person-first language (e.g., “individual who committed a sexual offence”) has begun to replace labels as a means to put the person before the behaviour. This language approach is intended to obstruct quick judgments associated with the ‘sex offender’ label, such as the stereotype that all individuals who commit sexual offences are alike – untreatable and at high risk of reoffending. Advocates argue that person-first language helps reduce stigma, fosters empathy, and emphasizes the importance of treating individuals with dignity and respect. Moreover, it provides opportunities for rehabilitation, reintegration, and the potential for desistance from crime.

The question we aim to answer is whether changing the labels used to describe individuals who have committed sexual offences can reduce negative perceptions and improve societal attitudes. While much research has examined the effects of labelling in other contexts (e.g., mental health), our study specifically focuses on whether person-first language can help foster more compassionate views toward individuals who sexually offend.

**Why was this study done?:**

The goal of this study was to compare the person-first language to various sex offender labels to examine if less stigmatizing language yields fewer negative perceptions about the individual, as well as whether perceptions would vary depending on the age of their victim. It is important to understand how language influences public perception because societal attitudes can significantly impact the rehabilitation of these individuals. For instance, if the public views individuals who have committed sexual offences more negatively, they may be less likely to support reintegration efforts or recognize the importance of rehabilitation programs. By understanding how different labels influence people’s views, the findings could inform policies or practices that aim to improve the reintegration of individuals into society in a more humane and supportive way.

**What did the researchers do and find?:**

In this experiment, the participants read one of eight different public announcement vignettes depicting a fictional individual who had committed a sexual offence. Four labels were used: person-first language, ‘sex offender,’ diagnostic labels (‘sexual sadist’ and ‘pedophile’), and offence-specific labels (‘rapist’ and ‘child molester’). Additionally, the victim’s age varied between adult and child. The contents of the news article were the same across the eight conditions, except for the label used to describe the perpetrator and the victim’s age. After reading the vignettes, participants were asked to rate their willingness to associate with the individual, the sentence severity that would be appropriately assigned to the perpetrator, the perpetrator’s likelihood to reoffend, and the perpetrator’s ability to be rehabilitated.

Contrary to expectations, the study found that the use of person-first language did not significantly reduce negative perceptions compared to more stigmatizing labels. Participants continued to endorse negative views, regardless of the label used, the victim’s age, or the interaction of the label and the victim’s age.

**What do these findings mean?:**

The current findings suggest that changing language alone may not be enough to change or influence societal attitudes toward individuals who sexually offend. While person-first language is an important step to ensure that individuals are treated with more humanity and respect, more comprehensive efforts may be necessary to address the underlying negative perceptions and biases that may detrimentally hinder reintegration.

## Highlights

- The study examines whether person-first language reduces negative perceptions about individuals who sexually offend.
- Labels used to describe the individual include person-first, ‘sex offender,’ diagnostic, and offence-specific labels.
- Participants continued to endorse negative perceptions, regardless of the label or victim’s age.
- Changing language alone may not be enough to change societal attitudes and perceptions toward those who sexually offend.

Individuals who have committed sexual offences are often portrayed in the media as high-risk, predatory, and incurable individuals (Galeste et al., 2012; Malinen et al., 2014). The media sensationalizes sexual offences by focusing on high-profile but rare cases, therefore creating an inaccurate representation of individuals who sexually offend (Lowe & Willis, 2022; Malinen et al., 2014). These portrayals have been associated with the use of the ‘sex offender’ label, depicting these individuals as a homogenous group despite the significant variations in their motivations, behaviours, and risks of reoffending (Galeste et al., 2012; Levenson et al., 2007; Willis, 2018). However, the use of the ‘sex offender’ label in the media, public policy, and research may impact decisions made by the public and decision-makers due to assumptions and attitudes associated with the label, resulting in decisions based on snap judgments rather than informed ones (Harris & Socia, 2016; Levenson et al., 2007; Malinen et al., 2014; Willis, 2018). Such labels may also negatively affect opportunities for rehabilitation, reintegration, and desistance (Willis, 2018). Within the context of promoting community safety through empirically informed policy, this paper evaluates the differences in public beliefs and opinions about individuals who have sexually offended as a function of the type of label used and the victim’s age. Specifically, the current study compares the person-first language to various sex offender labels to examine if less stigmatizing language yields fewer negative perceptions about the individual, as well as whether perceptions would vary depending on the age of their victim.

## Concerns With Criminal Label

Despite the adaptive function of labels in everyday communication, using these shortcuts to describe an individual can also cause harm (Willis, 2018). Labeling theory suggests that labeling individuals is counter-productive and can result in unintended consequences of influencing one’s identity and behaviour (Bernburg, 2019; Tannenbaum, 1938). For instance, research has found an association between the application of a deviant label and further deviancy and criminality (see Bernburg, 2019, for review of studies). Specifically, formal labeling of individuals who have engaged in criminal behaviour increases their risk for future reoffending, for example, through identity changes, blocked opportunities, and increased association with negative social influences (Bernburg et al., 2006; Restivo & Lanier, 2015; Wiley & Esbensen, 2016). Such labels might also be perceived as offensive, pejorative, and stigmatizing, potentially compromising an individual’s opportunities for rehabilitation, reintegration, and desistance (Willis, 2018). However, despite the potential for significant and damaging effects when stigmatizing labels are used, it is common that we rely on labels, perhaps as a form of distinguishing who is part of our ingroup and who is clearly seen as part of an outgroup, with whom there is less concern using stigmatizing or derogatory labels (e.g., Matsick et al., 2022).

## Public Opinion Research and Consequences of the ‘Sex Offender’ Label

The media often puts forth sensationalized and biased depictions of individuals who have sexually offended as being a high-risk, homogenous, and incurable population (Galeste et al., 2012; Malinen et al., 2014; Willis, 2018; Zatkin et al., 2022). There is published research that seem to show the public are likely to carry similar characterizations of these individuals (Harris & Socia, 2016; Levenson et al., 2007), and regrettably, this may result in their increased support for punitive actions but significantly reduced support for treatment and rehabilitation (Malinen et al., 2014; Willis, 2018). Unfortunately, these beliefs are contrary to the literature. Compared to those who perpetrate other criminal behaviours, research has found that individuals who have sexually offended have fewer criminal histories, lower recidivism rates, and vary in their risks of reoffending (Hanson et al., 2018; Lowe & Willis, 2020; Willis et al., 2010). Specifically, follow-up studies of adult males with a history of sexual offences have sexual recidivism rates of between 5% and 15% after five years and between 10% and 25% after ten years (Harris & Hanson, 2004; Helmus et al., 2012), and after 20 years, the rates are generally the same across risk levels (Hanson et al., 2024). It has also been found that treatment can significantly reduce an individual’s risk for future offending, and many individuals desist from committing crime (Olver et al., 2009; Willis et al., 2010).

Negative community attitudes towards individuals who sexually offend have been linked with greater support for policies that are more punitive, hinder access to treatment, and impair effective community integration (Cochran et al., 2021; Malinen et al., 2014; Willis, 2018). Research reveals that such policies usually involve longer periods of incarceration, registration, enhanced monitoring, public notification, and residence and employment restrictions (Kernsmith et al., 2009; Levenson et al., 2007; Mears et al., 2008). A more concerning finding is that respondents reported continued support for such policies even in the absence of reported effectiveness in reducing future offending behaviour (Levenson et al., 2007). Additionally, studies that did not frame perpetrators of sexual offences as a homogenous group found that respondents’ attitudes and support for particular policies depended on situational and offender characteristics. For instance, consistent with prior research (Ferguson & Ireland, 2006; Weekes et al., 1995), King and Roberts (2017) found that respondents displayed more punitive attitudes toward those who sexually offend in scenarios involving more serious offences, male offenders, older offenders, and younger victims, and consequently more deserving of harsh punishment and monitoring for the offender. Similarly, Kernsmith et al. (2009) examined the public support for registration for different types of individuals who sexually offend (e.g., incest, pedophile, date rapist). They found that respondents were more likely to support registration for individuals with child victims (97%) compared to statutory rape (65%). Regarding sentencing, Mears et al. (2008) found differences in the public’s support of incarceration for different forms of sexual offences. Over 90% of respondents supported incarceration for sexual assault against a child or an adult; however, there was less agreement among participants regarding indecent exposure and child pornography offences. Regardless, their findings indicate that sexual offences against a child were agreed upon as most deserving of incarceration.

Supporting these findings and other previous research (Cassidy & Rydberg, 2020; King & Roberts, 2017; Socia et al., 2021), a more recent study by Kruis et al. (2025) also found that participants recommended longer sentences, higher fines, and increased support for post-release sanctions for male perpetrators, older individuals, and those who committed violence against younger adolescents. Additionally, Calobrisi and Knight (2024) examined how the age of the victim influences the public’s risk assessment and punishment attitudes for individuals who have sexually offended. As predicted, they found that risk ratings for those who sexually offended against children were significantly higher than ratings for adult victim vignettes. Consequently, participants assigned more punitive dispositional placements for those with child victims than for those with adult victims, regardless of risk level. Overall, these studies reflect how entrenched public attitudes about the dangerousness of these individuals are, especially when they sexually perpetrate against children.

## Person-First Language and the Mental Health Field

In the 1990s, the movement toward person-first language emerged out of the concern that labels promote bias, devaluation, and negative attitudes toward the individual (American Psychological Association [APA], 2020). The Americans with Disabilities Act of 1990 developed guidelines for the proper use of person-first language in recognition that using labels to define individuals resulted in greater stigmatization across medical, legal, and social domains (McCoy & DeCecco, 2011; Russell et al., 2005). The *Publication Manual of the American Psychological Association* (APA, 2020) advocates for possessive, postmodified language (e.g., person with schizophrenia) over premodified or noun-based labels (e.g., schizophrenic) to separate the individual from their diagnosis or condition, where the diagnosis is no longer their sole defining characteristic (APA, 2020; Crocker & Smith, 2019; Jensen et al., 2013). Willis (2018) also indicates that using person-first language respects the client’s dignity and benefits their welfare.

In a study conducted by Reynaert and Gelman (2007), they examined the effect of linguistic form (i.e., noun, adjective, and possessive phrases) on judgments of the permanence of mental and physical illness. They found that in the mental illness condition, the noun phrase “is a” was perceived by undergraduate students as more permanent when compared to adjective and possessive phrases. Moreover, there is evidence to suggest that people’s attitudes differ on account of a label. For instance, Granello and Gibbs (2016) administered the Community Attitudes Towards the Mentally Ill to undergraduate students, counsellors, and community samples. Half of the participants were administered the survey using a noun label and the other half completed a version of the survey using a person-first language. Across all three samples, they found that individuals who were assigned to the person-first condition reported lesser stigmatizing attitudes than those in the noun label condition. However, not all research encourages the use of person-first language. Botha et al. (2022) investigated the perspectives of autistic research participants and found that participants were more inclined to reject person-first labels, preferring to reclaim the language and using identity-first language, which self-designates themselves as autistic.

## Person-First Language, the ‘Sex Offender’ Label, and Decision Making

In adhering to the APA guidelines (2020) that advocate for the use of neutral terminology and following in line with the changes established in other fields of psychology, the use of noun-based labels is becoming less acceptable. For instance, the *Sexual Abuse* journal of the Association for the Treatment and Prevention of Sexual Abuse encourages using more respectful language to describe individuals and groups in manuscript submissions. Preferred terminologies include “individuals who commit sexual offences” or “person with sexual interest in prepubescent children,” as opposed to terms like “sex offender” or “pedophile,” respectively. Using these terms is suggested to reduce bias and is assumed to improve opportunities for rehabilitation, reintegration, and desistance from crime (Willis, 2018).

Previous research suggests that the “sex offender” label reinforces the negative misconceptions about individuals who perpetrate sexual violence (Harris & Socia, 2016). Stronger punitive attitudes have been associated with how closely an individual matches the ‘sex offender’ schema (i.e., a stereotypically incurable, predatory, and highly dangerous offender; Galeste et al., 2012; Harris & Socia, 2016). Stigmatizing labels are thought to lead to heuristically based decision-making. A heuristic is a cognitive shortcut that enables quick, intuitive judgments based on past associations. One type of heuristic is the availability heuristic (Hjeij & Vilks, 2023; Tversky & Kahneman, 1973), wherein individuals make decisions based on readily available information. For instance, due to the media’s fixation on high-profile sexual offences, the term ‘sex offender’ is readily associated with the constructed stereotypical image of a violent, predatory male individual despite the fact that only a few offenders meet this description (Quinn et al., 2004). Similarly, the representativeness heuristic (Hjeij & Vilks, 2023; Tversky & Kahneman, 1974) suggests that judgments are often based on how closely something resembles previous experiences. The media perpetuates the image of a population of those who sexually offend that is collectively high-risk and untreatable (Galeste et al., 2012). Therefore, it is likely that the public uses this knowledge to make judgments about the whole population. The last relevant heuristic is the affect heuristic (Slovic et al., 2007), which refers to judgments based on prior emotional associations. The ‘sex offender’ label elicits emotional reactions of fear or disgust that can influence the public’s perceptions about them (Galeste et al., 2012).

Person-first language is intended to obstruct the snap judgments that the ‘sex offender’ label evokes, such as the stereotype that all persons that commit sexual offenses are the same. This is known as the myth of homogeneity, which suggests that all individuals who sexually offend are untreatable and at a high risk to reoffend (Galeste et al., 2012; Levenson et al., 2007). In turn, using person-first language will allow for more informed and neutral decision-making that considers the diversity within and between groups of individuals who sexually offend (Harris & Socia, 2016). A few studies have demonstrated that punitive attitudes can differ as a function of the type of label used. For instance, Harris and Socia (2016) found that when participants were asked to rank their agreements on a series of statements about individuals who sexually offended, those in the “sex offender” and “juvenile sex offender” label conditions supported more punitive policies (e.g., registries and residence restrictions) than those in the neutral language condition. Imhoff (2015) found a similar trend, where there were more stigmatizing and punitive attitudes associated with “pedophile” than more descriptive terms such as “people with sexual interests in prepubescent children.” Consistent with prior research (Harris & Socia, 2016; Imhoff, 2015), Lowe and Willis (2020) also found that compared with neutral descriptors, labels were linked with less willingness to volunteer with those who have sexually offended. However, a study conducted by Snape and Fido (2022) found that the labels assigned to people with sexual offences had no significant relationship with public perceptions. Specifically, individuals held similar perceptions of an offender regardless of whether they were referred to as ‘sex offenders’ or ‘persons with sexual convictions.’

Additionally, research has found that the public and media often view the paraphilia of sexual sadism or pedophilia as being synonymous with acts of sexual violence (e.g., rape or child molestation), but the sexual interest can be present in the absence of offending behaviour (Feelgood & Hoyer, 2008; Imhoff, 2015; Jahnke et al., 2015; Kirsch & Becker, 2007). Regardless of this, pedophilia in the absence of any criminal behaviour is associated with similar levels of stigmatizing and punitive attitudes (Imhoff, 2015; Jahnke et al., 2015). Despite these findings, a study conducted by Martijn et al. (2020) found that individuals who reported having sexual attraction toward children were more likely to endorse terms such as child lover, pedophile, and minor attracted person, as opposed to person-centred language. In a more recent study, Jahnke et al. (2022) investigated the attitudes and preferences regarding ‘pedophile’ and other labels among 286 individuals who reported a strong sexual attraction to children. Using a mixed-method study, they found that, consistent with Martijn et al. (2020), these individuals did not prefer the use of person-first language, as it implied that pedophilia is undesirable and innately pathological. When asked about their preferred label, only a small number of participants indicated person-first language, while the majority preferred the terms ‘minor-attracted person’ or ‘pedophile/hebephile’ (Jahnke et al., 2022). Overall, these studies demonstrate that although prior research on public opinions and attitudes towards perpetrators of sexual violence may be influenced by the labels used to describe the individual, these labels may not necessarily be problematic to those who identify with the labels.

## The Current Study

Broadly, the current study examined the effect of using stigmatizing labels to describe individuals who commit sexual offences. The field of mental health has adopted the use of person-first language because it has been argued that its use would lessen the stigma and discrimination towards persons with mental illness (APA, 2020; Reynaert & Gelman, 2007). Going off the similar premise of separating the individual from their illness, this study examines whether separating the individual from their criminal act would have the same outcome of reducing stigma and discrimination. Specifically, this study assessed whether using person-first language could result in less negative perceptions and more informed decisions about a fictitious individual being released into the community after serving a sentence for a sexual offence conviction. Using public announcement vignettes, we evaluated the public’s willingness to associate and preferred severity of the sentence, as well as the public’s view on the perpetrator’s perceived social normality, perceived dangerousness, and treatment amenability. It was predicted that individuals in the person-first language condition would have fewer negative perceptions and respond less punitively and in a less stereotypically consistent manner because it does not evoke the ‘sex offender’ schema, which would align with prior research (e.g., Harris & Socia, 2016). In contrast, those who read narratives that used stigmatizing labels, such as ‘sex offender,’ diagnostic terms (e.g., pedophile), or offence labels (e.g., child molester) would respond more negatively. Additionally, we assessed whether the respondent’s perceptions about individuals who have sexually offended would vary depending on the age of their victim. Consistent with previous literature that has found that sexual offences against children were viewed more negatively compared to those with adult victims (e.g., Calobrisi & Knight, 2024; King & Roberts, 2017; Kruis et al., 2025), it was hypothesized that individuals in the child victim conditions would respond negatively overall, regardless of the label used.

## Method

### Sample

Four hundred and ninety-three participants were recruited for the present study. To be eligible, participants had to be at least 18 years old, reside in Canada or the United States, and be able to complete the study in the English language. We excluded participants who did not meet these criteria, as well as those who did not complete a sufficient number of survey questions (i.e., less than 75% of the survey; 21 participants), spent too little time completing the study (i.e., less than five minutes or more than two hours; 73 participants), or incorrectly reported key elements of the presented news articles (e.g., crime, victim’s age; 107 participants). As a result, a total of 297 participants were included in the final analyses. This final sample consisted of 55.6% males (*n* = 165), with an average age of 33.3 years (*SD* = 9.89) and ranged from 19 to 76 years. The majority of the participants were from Canada, and 10.4% (*n* = 31) reported living in the United States. With regards to educational attainment (97.3% response rate), 18.2% (*n* = 54) reported having either some high school or completed high school education, 25.2% (*n* = 75) reported either being in the process of or completing a trade or college diploma, 39.4% (*n* = 117) reported either being in the process of or completing a bachelor's degree, and 14.5% (*n* = 43) reported either being in the process of or completing a masters or doctoral degree. When asked if they were a parent (99.3% response rate), 56.9% (*n* = 169) reported being a parent, while 42.4% (*n* = 126) reported not being a parent. Regarding previous experience with sexual violence, 12.8% (*n* = 38) of participants disclosed having experienced it themselves, 31.7% (*n* = 94) knew someone who experienced it, and 24.6% (*n* = 73) knew someone who had committed a sexual offence.

### Materials

#### Vignette

Participants were asked to read a newspaper community notification vignette to examine the role of various labels on judgments of social distance, treatment amenability, risk of recidivism, and sentencing severity. All participants were randomly assigned into one of the eight conditions: 4 labels (person-first, ‘sex offender,’ diagnostic, or offence-specific labels) by 2 victim’s ages (committed against an adult or child). The diagnostic labels included ‘sexual sadist’ and ‘pedophile,’ while the offence-specific labels included ‘rapist’ and ‘child molester.’ All vignettes described the release of a fictitious individual convicted of a sexual offence back into the community. The contents of the news article were constant across the eight conditions, except for the label used to describe the perpetrator (found in the headline and body of the news article) and the victim’s age (indicated in the body of the news article). Additionally, there was minimal information about the sexual offence provided in the vignette, so the differences produced could only be attributed to the label rather than the content (see Appendix for the full vignette announcements).

#### Dependent Measures

##### Social Distance and Anticipatory Behaviour

Participants were administered two measures assessing their opinions regarding differing social distances from the individual depicted in the vignette. The first scale was a 11-item self-report questionnaire adapted and modified by Malinen et al. (2014) from Bogardus’ (1925) Social Distance Scale (SDS). These items measured the participant’s willingness to have a newly released individual who committed a sexual offence as their neighbour, employer, close friend, or son-in-law. This revised measure by Malinen et al. (2014) demonstrated excellent internal consistency (α = .95). This questionnaire used a horizontal 100-point scale ranging from 0 (*definitely not*) to 100 (*definitely yes*), which varied from the scale utilized by Malinen et al. (2014; ranging from *most definitely* to *definitely not*) to ensure consistency throughout the other measures. Therefore, higher scores indicate more positive attitudes toward individuals depicted in the newspaper.

The second scale consisted of seven items from the Social Distance subscale of the Attitudes Toward Sex Offenders Scale–21 (ATS–21; Hogue et al., 2019) and further assessed how socially distant individuals who have sexually offended appear to the public. Original items were scored using a 5-point Likert scale; however, this was modified to a 6-point scale ranging from 1 (*strongly disagree*) to 6 (*strongly agree*) in the present study to incorporate with the items assessing treatment amenability and keep the responses consistent across the dependent measures. The ATS-21 demonstrated high internal consistency (α = .91; Hogue et al., 2019). These items were summed independently of the treatment amenability items in the scale to produce a single social distance score. Higher ratings indicate more positive attitudes or greater tolerance of the released individual in the community.

##### Risk to Reoffend

Participants were asked to rate how likely the individual depicted in the newspaper would reoffend with: 1) the same type of offence (sexual crime); and 2) any type of offence (sexual or non-sexual crime). Both items were rated on a scale from 0% to 100%. A higher reported percentage is indicative of a stronger belief that the individual will reoffend when released in the community and is related to perceptions of greater dangerousness.

##### Sentencing and Management

On a scale from 1 to 10, participants were asked three questions related to the sentencing and management of the individual in the news article. The first question asked the participants if they would have proposed a sentence that was more rehabilitative (0), a balance of rehabilitation and punishment (5), or more punitive (10). The second item relates to the level of supervision the individual should receive upon release into the community, ranging from no supervision (0), moderate supervision (5), to close monitoring (10). In the last question, participants were asked to rate the intensity of the treatment (low, moderate, or high) the individual should receive while in the community. Higher whole-scale scores for this measure indicate stronger punitive beliefs about the individual convicted of a sexual offence.

##### Treatment Amenability

Seventeen items were taken from the Community Attitudes Towards Sex Offenders Scale (CATSO; Church et al., 2008) and the Attitudes Towards the Treatment of Sex Offenders (ATTSO; Wnuk et al., 2006) scales to address a participant’s perceptions of how well the individual depicted in the vignette will respond to treatment. Specifically, three subscales were used but modified for this study, and included the Capacity to Change subscale from the CATSO and the Treatment Ineffectiveness and Incapacitation subscales from the ATTSO. The CATSO demonstrated adequate internal reliability (α = .74; Church et al., 2008), whereas the ATTSO showed high internal consistency (α = .86; Wnuk et al., 2006). The current study utilized a 6-point Likert scale (1 – *Strongly Disagree*, 6 – *Strongly Agree*) as opposed to the subscales’ original 5-point scale, making the questionnaire a forced-choice response by removing the *Undecided* option. Participants in the pilot study expressed reluctance to respond to questions because of the lack of information about the individual in the vignette. Therefore, this change was grounded in the hope that the forced choice would indicate the direction of the participant’s response. Higher scores on this measure indicate the participant’s belief that the individual is untreatable.

### Procedure

The present study received ethics approval from the authors’ university to conduct a pilot study and the main study. The study was piloted with 81 undergraduate students who were recruited from a university introductory psychology research pool comprising students who completed studies for 2% course credit towards their overall course grade. The majority of the student sample (92.6%) were between the ages of 18 and 23, and a small number (*n* = 6) were between 24 and 35 years of age. Data from the pilot student sample was used to improve the survey before administering it to the broader community population and was not included in formal analyses.

For the main study, community adult participants were recruited and compensated via Crowdflower. The crowdsourcing platform Crowdflower (http://www.crowdflower.com/) is an online research platform where participants complete human intelligence tasks, such as research surveys, for monetary compensation. In a study comparing Crowdflower to other platforms, including Amazon Mechanical Turk (MTurk), Crowdflower participants provided higher response rates, demonstrated the highest ethnic diversity, had a lower propensity to engage in dishonest behaviour, and were more naïve regarding knowledge about experimental methods compared to MTurk (Peer et al., 2017). However, Peer et al. (2017) found that Crowdflower participants were less attentive to the questions as measured by failed attention check questions, resulting in lower internal reliability. Hence, this study included manipulation checks.

The present study was administered using Qualtrics survey software. All participants were informed of the study details, benefits, and risks of participating, and asked for their consent to proceed. Participants were free to withdraw at any point of the study without loss of compensation. Participants were initially told that the study looked at community attitudes towards individuals who have committed sexual violence and sentencing decisions. After completing the consent form, participants were randomly assigned to one of eight conditions, where they were asked to read a news article depicting the release of a fictitious individual who was convicted of a sexual offence back into the community. The contents of the news article were constant across conditions, except for the independent variables, which varied the perpetrator label and the victim’s age.

Following the presentation of the vignette, participants completed post-manipulation check questions to check whether participants correctly reported that the individual in the vignette was not a murderer (i.e., ensure they attended to the offence in the vignette) and whether they correctly identified the victim’s age (i.e., ensure the age variable was salient). Those who responded correctly were included in the final sample. Then, participants were presented with a series of dependent measures related to the individual depicted in the newspaper. The presentation of these measures was counterbalanced. Participants were also asked to complete a series of questions about demographic information, their experience of sexual violence, and if they knew someone who had been affected by or perpetrated sexual violence. All participants were compensated with $0.50 CAD, irrespective of survey completion. After completing the survey, participants were given a short explanation of the study’s purpose and the variables of interest, as well as a list of mental health resources and the researchers’ contact information.

## Results

Statistical analyses were carried out using SPSS version 28. Two multivariate analyses of variance (MANOVAs) were conducted with the two independent variables, the type of label used to describe the individual who committed a sexual offence (four conditions) and the age of the victim (two conditions). The first MANOVA was conducted for the six dependent measures, which included two scales that assess willingness to associate, belief in treatment non-amenability, perceived dangerousness for sexual and general reoffending, and sentencing severity. The second MANOVA was conducted for the three subscales of the treatment amenability scale. A *p*-value of .05 was used to determine statistically significant outcomes. Correlations among the dependent variables are mostly significant and ranged from -.241 to .667, as seen in Table 1, except for a weak to negligible non-significant correlation between the Social Distance Scale and the full scale of treatment amenability (*r* = -.059). The means and standard deviations for each dependent variable across the four label conditions and two victim age conditions are listed in Table 2.

**Table 1:** Intercorrelations of Dependent Variables

Measures	1	2	3	4	5	6	7	8	9
1. Social Distance Scale	–	–	–	–	–	–	–	–	–
2. ATS-21 Social Distance	.551***	–	–	–	–	–	–	–	–
3. Treatment Amenability	–.059	–.292***	–	–	–	–	–	–	–
4. Capacity to Change	–.138*	–.280***	.827***	–	–	–	–	–	–
5. Treatment Ineffectiveness	–.208***	–.439***	.652***	.390***	–	–	–	–	–
6. Incapacitation	.056	–.156**	.907***	.630***	.410***	–	–	–	–
7. Sexual Recidivism	–.432***	–.340***	.381***	.404***	.201***	.323***	–	–	–
8. General Recidivism	–.319***	–.241***	.327***	.357***	.133*	.274***	.667***	–	–
9. Sentencing Severity	–.355***	–.259***	.204***	.320***	.129*	.111	.666***	.523***	–

*Note. N*s = 232 to 295. Pearson correlation coefficients are reported. ATS-21 Social Distance = Social distance items modified from the Attitudes Toward Sex Offenders Scale-21.

*p < .05. **p < .01. ***p < .001.

**Table 2:** Descriptive Statistics of Dependent Variables Across Four Label Conditions and Two Victim Age Conditions

Dependent Measures	Person-First		“Sex Offender”		Offence-Specific		Diagnostic	
	Child (*n* = 33)	Adult (*n* = 35)	Child (*n* = 32)	Adult (*n* = 43)	Child (*n* = 37)	Adult (*n* = 39)	Child (*n* = 39)	Adult (*n* = 39)
1. Social Distance Scale	309.0 (228.62)	378.7 (357.75)	259.2 (266.89)	371.9 (284.49)	336.1 (260.64)	256.1 (223.95)	290.1 (252.42)	408.2 (271.58)
2. ATS-21 Social Distance	22.6 (5.80)	24.0 (6.09)	23.3 (6.52)	24.3 (6.55)	23.2 (4.61)	23.1 (6.09)	23.6 (6.00)	24.9 (6.40)
3. Treatment Amenability	56.2 (13.52)	60.8 (12.92)	58.6 (11.43)	58.3 (13.95)	56.2 (9.59)	61.9 (14.90)	60.4 (11.82)	59.7 (14.26)
4. Capacity to Change	16.8 (4.84)	19.2 (4.32)	17.0 (4.47)	17.2 (4.46)	18.3 (4.00)	18.3 (5.31)	18.3 (4.46)	17.8 (4.86)
5. Treatment Ineffectiveness	13.8 (3.49)	13.6 (3.80)	14.1 (3.82)	14.5 (4.10)	13.2 (3.26)	15.6 (3.76)	14.1 (3.40)	14.0 (3.96)
6. Incapacitation	25.0 (7.72)	27.7 (7.39)	26.8 (7.35)	26.8 (8.37)	25.4 (5.43)	27.7 (7.42)	27.7 (7.40)	27.1 (8.47)
7. Sexual Recidivism	64.7 (21.01)	61.0 (24.12)	62.7 (20.81)	58.9 (22.99)	59.8 (23.42)	58.1 (24.03)	61.3 (22.97)	57.3 (22.33)
8. General Recidivism	57.1 (24.52)	58.7 (23.36)	60.6 (25.73)	50.8 (24.91)	60.9 (22.83)	57.6 (21.53)	57.3 (25.28)	57.3 (23.99)
9. Sentencing Severity	19.9 (5.92)	19.2 (6.24)	20.8 (6.73)	19.4 (5.60)	20.1 (5.19)	20.4 (5.61)	19.7 (6.99)	18.4 (5.53)

*Note.* Means and standard deviations (in parenthesis) are listed. *N*s = 252 to 296. Social Distance Scale (0 – 1100) where lower scores indicate a lesser desire to associate with the individual convicted of a sexual offence. Social Distance from the ATS-21 (7 – 42) where lower scores indicate a greater social distance from the individual convicted of a sexual offence. Treatment Amenability (17 – 102) consists of 3 subscales: Capacity to Change (5 – 30), Treatment Ineffectiveness (4 -24), and Incapacitation (8 – 48). Higher scores on the Treatment Amenability scale and subscales indicate negative perceptions about the person’s treatability, capacity to change, treatment effectiveness, and need for incapacitation. Recidivism (0% – 100%) where higher scores indicate a greater probability that the person will reoffend. Sentencing Scale (3 – 30) where higher scores indicate stronger punitive beliefs about the individual convicted of a sexual offence.

The assumptions of a MANOVA analysis, including the normality of the residuals and the absence of multivariate outliers, were checked for all the models. Further, since the sample sizes for all eight experimental conditions were approximately the same, we did not test for the assumption of equal variance matrices. Pillai’s Trace was selected as the MANOVA’s test statistic, given its robustness towards a violation of equal covariance matrices. Line graphs, including error bars (± 2*SE*), were employed to illustrate the association between each dependent variable and each independent variable (i.e., the label type and victim’s age).

To test the central hypothesis that person-first language will yield significantly less negative and punitive responses regarding individuals who have sexually offended, the first MANOVA was conducted on our six main dependent variables. Our findings revealed that there was no statistically significant difference in social distance, treatment amenability, sexual recidivism, general recidivism, and sentencing severity based on the label type, *F*(18,627) = .549, *p* = .934; Pillai’s Trace = .047, partial η^2^ = .016, the victim age, *F*(6,207) = 1.993, *p* = .068; Pillai’s Trace = .055, partial η^2^ = .055, or their interaction, *F*(18,627) = .085, *p* = .435; Pillai’s Trace = .085, partial η^2^ = .028. The assumptions for this model were met to an acceptable degree. Refer to Figure 1 for the line graphs of the main dependent variables by the type of label used and the victim’s age.

A second MANOVA was conducted on the three subscales of the treatment amenability scale measuring the incapacity to control sexual impulses, treatment ineffectiveness, and incapacitation. Our findings revealed that both the main effects and the interaction of the label type and victim’s age did not have statistically significant effects on any of the amenability subscales. Specifically, there was no statistically significant difference in the capacity to change, treatment ineffectiveness, and incapacitation subscales based on the label type, *F*(9,798) = .917, *p* = .510; Pillai’s Trace = .031, partial η^2^ = .016, victim age, *F*(3,264) = .009, *p* = .514; Pillai’s Trace = .009, partial η^2^ = .009, or their interaction, *F*(9,798) = 1.553, *p* = .125; Pillai’s Trace = .052, partial η^2^ = .017. Similarly, the assumptions for this model were also found to be met to an acceptable degree. See Figure 2 for the line graphs of the three subscales of the treatment amenability scale.

**Figure 1:**
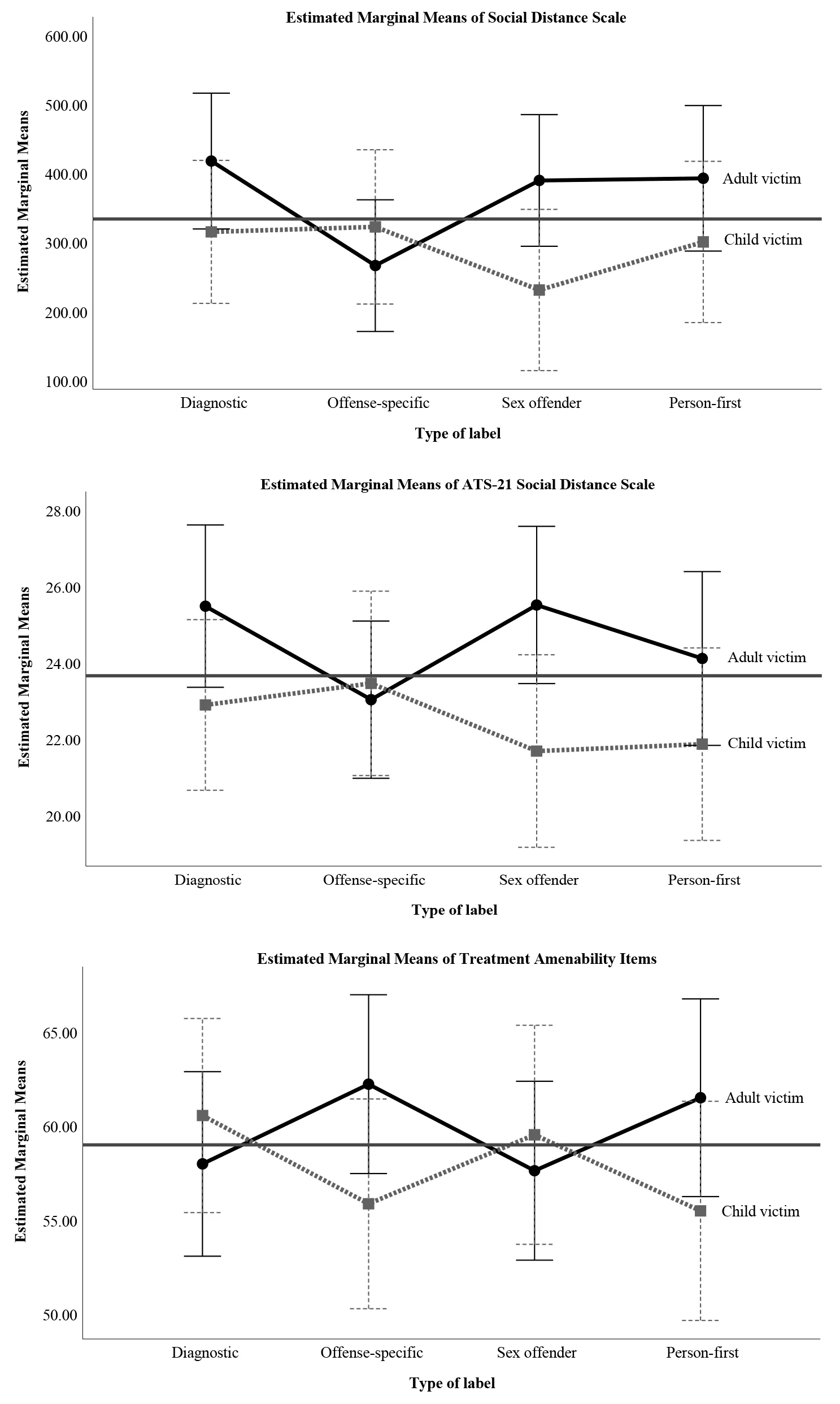
Line Graphs of the Six Main Dependent Variables by the Type of Label and Age of the Victim *Note.* The center dark gray horizontal line refers to the grand observed mean for the sample.

**Figure 1 (continued):**
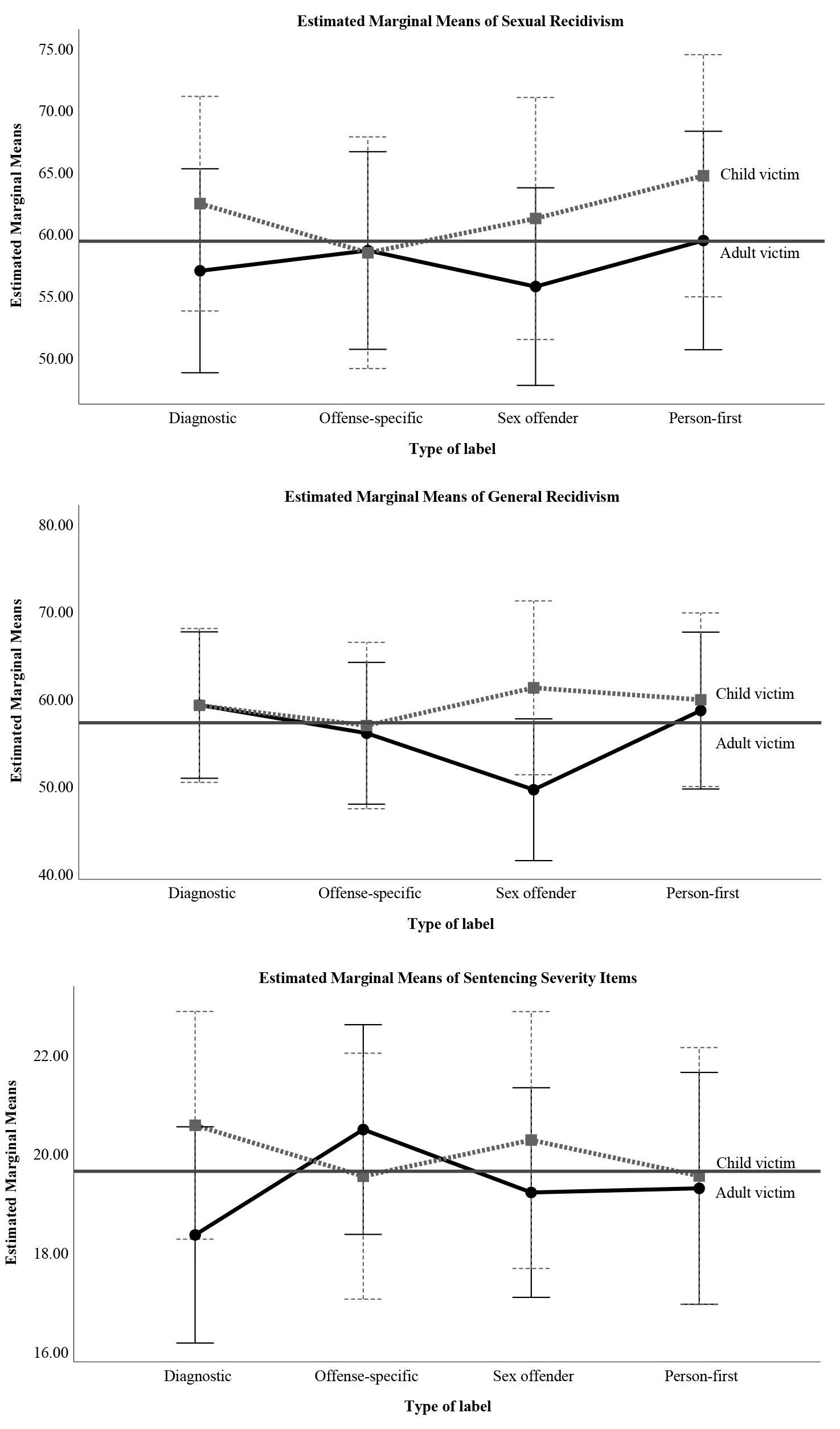
Line Graphs of the Six Main Dependent Variables by the Type of Label and Age of the Victim *Note.* The center dark gray horizontal line refers to the grand observed mean for the sample.

**Figure 2:**
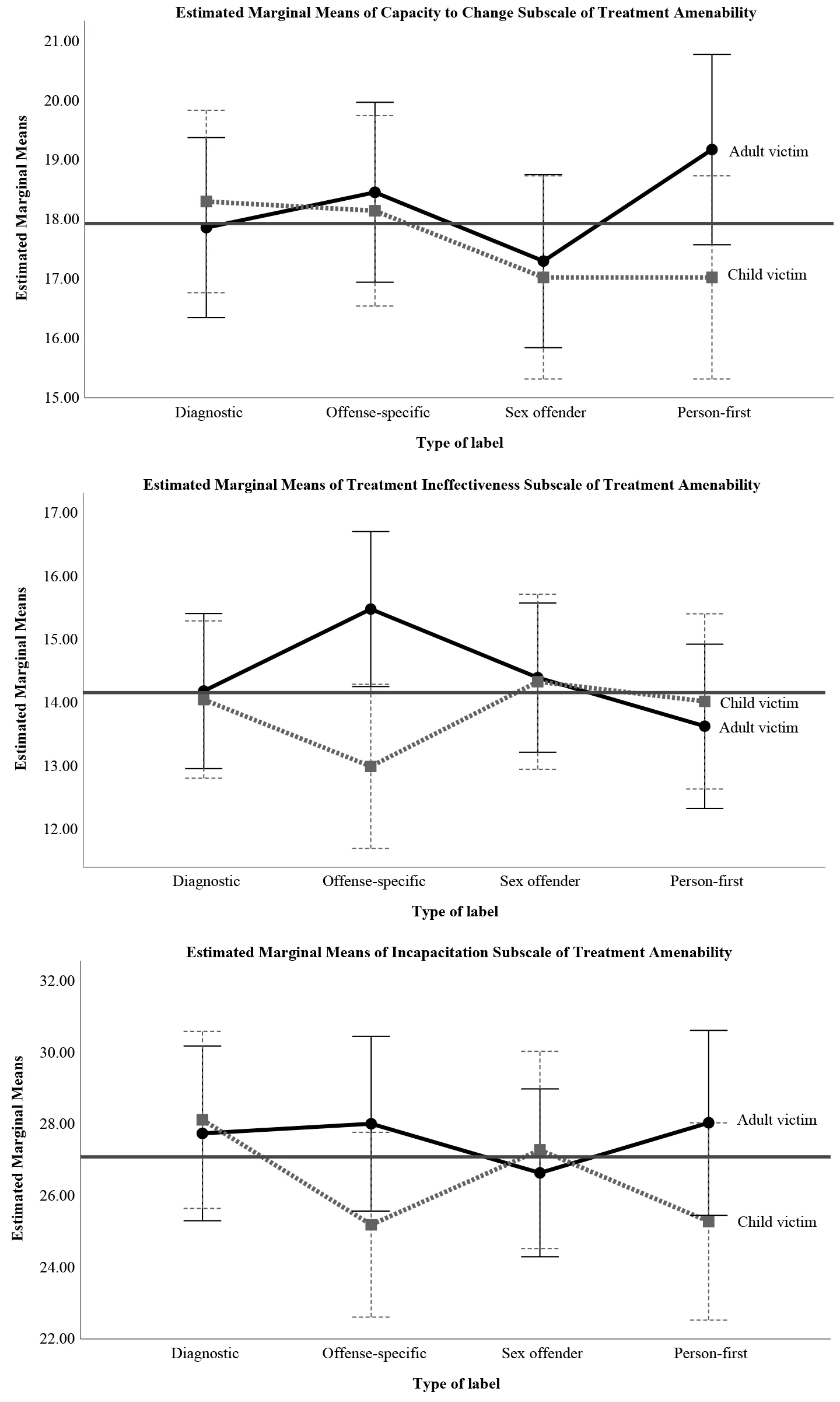
Line Graphs of the Treatment Amenability Subscales by the Type of Label and Age of the Victim *Note.* The center dark gray horizontal line refers to the grand observed mean for the sample.

## Discussion

The current study examines whether using different language to describe individuals who sexually offend would make a difference in the perceptions of community members of those individuals. Specifically, person-first language and three stigmatizing labels (i.e., generic, diagnostic, and offence-specific) were varied to examine public perceptions towards a fictional adult male who sexually offended against a child or an adult victim. Borrowing from research that has demonstrated reduced stigmatizing perceptions when person-first language is used to describe those with a mental disorder, many researchers in the field of sexual violence prevention have suggested the same effect would result when changing language that describes individuals who sexually offend – that is, using person-first language may reduce stigmatizing attitudes in the context of those who have perpetrated sexual offences (e.g., Lowe & Willis, 2020, 2022).

In contrast to these views, our experimental study did not produce findings that support this conclusion, despite further varying the types of labels often used in the media and community notifications by including diagnostic (i.e., sexual sadist, pedophile) and offence-specific labels (i.e., rapist, child molester), in addition to the general label of ‘sex offender.’ Our results align with other empirical research that does not consistently substantiate this conclusion (e.g., Harris & Socia, 2016), including a recent study conducted in the United Kingdom that concluded the label assigned to people with sexual offences had no significant relationship with public perceptions (Snape & Fido, 2022). Additionally, consistent with the findings of Martijn et al. (2020), Jahnke et al. (2022) found that the majority of the individuals who reported a strong sexual attraction to children preferred to use the terms ‘minor-attracted person’ or ‘pedophile/hebephile’ rather than person-first language. These individuals also expressed a preference for embracing their sexuality as part of their identity and desired this perspective to be reflected in the professional discourse. Although other research has shown differences in how the public perceives those who sexually offended against a child versus an adult (e.g., usually viewing those who sexually offend against children more negatively, as less amenable to treatment, and more at risk to reoffend sexually; Calobrisi & Knight, 2024; Jung et al., 2012), our analyses were not sensitive to capture any differences. The designs of these previous studies differed from the current study (e.g., Calobrisi & Knight’s study presented 5 separate vignettes to each participant that either included child victims or adult victims), and therefore, the salience of our experimental manipulation may not have been as salient to participants.

Improving our language by eliminating stigmatizing labels is a positive initiative and, as noted, is a humane approach endorsed by APA, which emphasizes person-first over identity-first language (APA, 2020). However, singling out language as an initiative for change in this context may be somewhat presumptive. In fact, most studies, ours included, have ceiling effects and consistently show negative perceptions about individuals who sexually offend, especially about those who sexually offend against children (Jung et al., 2012). The pattern of responding among the participants in this study is consistent with general literature on public perceptions of sexual offending that suggests participants endorse the myth of homogeneity; that is, they view the entire population of persons who sexually offend as the same across offender characteristics, such as being highly likely to reoffend again and being untreatable (Galeste et al., 2012; Katz-Schiavone et al., 2008; Levenson et al., 2007; Magers et al., 2009).

In more general research that examines public perceptions of released individuals with violent convictions in the context of community notifications, varying the language and content of the notification did not change the public’s tolerance for the individual, the belief that the individual would benefit from treatment, and views about the individual’s likelihood to reoffend (Himmen et al., 2023). Instead of expecting that language may influence public perceptions, greater attention should focus on other initiatives to reduce stigmatization. For instance, Spivey (2024) calls for a broader conversation about stigma resistance that involves rejecting, reframing, and refocusing strategies. Further, it is undoubtedly the case that those who sexually offend experience first-hand how stigma impacts their re-entry into the community and their success at leading a prosocial existence in society, but the question of whether the label itself will make a difference is not consistently supported empirically.

A recent study by Belisle et al. (2025) used a mixed-methods approach to assess whether the use of person-centred language in a classroom context would affect students’ perceptions of individuals who have committed sexual offences. Their study included three experimental course conditions: a course section where no person-centred language was used, a section where the professor incorporated person-centred language in all course materials, and a section where person-centred language was combined with a guest lecture on its nature and importance in criminal justice settings at the beginning of the course. Their findings indicated that person-centred language alone did not significantly impact student perceptions. However, when person-centred language was combined with the informational lecture, there were significant changes in students’ attitudes and perceptions regarding the sentencing and management of individuals who perpetrate sexual offences. No significant differences were observed for the stereotype endorsement or risk perception subscales, although participants in this condition showed the greatest mean reduction in perceived risk. Qualitative data further indicated that these changes reflected more positive opinions on rehabilitation, a belief in the potential for change, and a better understanding of recidivism rates among this population. Overall, this study suggests that simply using person-centred language may not be enough to influence perceptions. Instead, providing a clear and thorough explanation of its rationale – emphasizing the humane treatment of the individuals in this population and how labels can contribute to stigmatization and bias – appears necessary to bring about meaningful changes in perceptions of sentencing, management, and potentially the risk posed by individuals who commit sexual offences. However, that being said, a control condition that included a lecture on stigmatizing language without using person-centered language was not a condition of the study. Furthermore, research has yet to examine the long-term effects of how sustained language change (i.e., one’s consistent use of person-first language to describe those with disabilities, mental disorders, proclivity to sexually offend, etc., in all contexts) may allow one to adapt and evolve in their attitudes and potentially have an impact in altering their behaviour.

The implications of changing our language when describing individuals who sexually offend (e.g., media, community notifications, written reports) or when working with these individuals (e.g., treatment, supervision, parole hearings) may not directly change public perceptions but, in essence, it can facilitate more humane treatment. Furthermore, it aligns better with trauma-informed and compassionate approaches to promote positive and prosocial changes among individuals who sexually offend (Levenson et al., 2020). Such approaches also address responsivity issues, which can be obstacles for justice-involved individuals to engage in rehabilitation or supervision (Jung, 2022). When one uses pejorative labels, such as ‘sex offender,’ or diagnostic labels personifying the individual, such as ‘pedophile,’ it runs counter to rehabilitative efforts (Willis, 2018). In addition to those in clinical or supervision positions, the media and other public bodies, including politicians and stakeholders, have a responsibility to use humane descriptions of the individual, and such compassion should be encouraged and modelled, despite what society may feel about these individuals. Further, we should recognize and acknowledge that the public will continue to see most individuals who sexually offend, especially those who offend against children, in a negative and stigmatizing light, regardless of what label we use (e.g., Jung et al., 2012). Hence, using person-first language is important to ensure we treat all people with respect, including those who have conducted themselves egregiously. However, in light of the current study’s findings, we should not expect that changing language alone will change societal attitudes or perceptions.

There are important limitations to take into consideration that could have impacted the quality and generalizability of the data in this study. Participants were recruited through online crowdsourcing, which allowed for a more diverse sample than given by a university sample; however, it cannot be determined whether the non-significant findings could be attributed to a lack of attention, which is a common issue with using online studies. Given that our study is experimental in nature, it allowed us to control the content and focus on the language manipulation that we were interested in but may have reduced realism. Although we attempted to ensure a degree of realism by presenting cases in the form of newspaper articles from a well-known national newspaper rather than using hypothetical cases or questions about sexual offenders in general, as seen in previous studies, the salience of the language manipulation may not be as prominent and may have limited the chance to find real differences. Furthermore, we used labels that may be characterized as diagnostic in nature, such as ‘pedophile’ in the current study; however, it is important to note that media also often employ these diagnostic terms, sometimes inappropriately. Additionally, it is a limitation that some of the person-first language used in the vignettes may not align with the stigmatizing labels included (e.g., comparing “sexual sadist” with “rapist”). Therefore, further research may consider aligning these terminologies to facilitate better comparison. Lastly, our measures that focus on sentencing comprised of three questions pertaining to scales for rehabilitation and punishment, supervision level, and treatment intensity, did not account for what we know with regards to the principles of effective rehabilitation (otherwise known as the risk, need, and responsivity principles; Bonta & Andrews, 2024).

In terms of future directions, it is important to note that our study only focused on public perceptions of men who sexually offend. Hence, our conclusions cannot be generalized to the perceptions of women. Studies on perceptions of the community on women who perpetrate sexual offences have mostly focused on general perceptions rather than the use of language or labeling (e.g., Zack et al., 2018), and therefore, further research is needed to examine whether person-first language would have an impact on changing attitudes towards women. Similarly, our findings can only be generalized to adults and may be unlikely applicable to youths who sexually offend. In Harris and Socia’s study (2016), more pronounced and robust differences were found in the endorsement of restrictive policies and practices when it came to the language used to describe juveniles who sexually offend, suggesting that perhaps the lack of support for changing language may only be applicable to perceptions of adults who offend and not youths. Related to the lack of findings, although we had nearly equivalent samples sizes for all of the eight conditions, the overall power may not be enough to detect a difference or an interaction, and therefore, future studies should include a larger sample of community participants.

Another consideration to note is that using student samples and community-recruited samples or participants recruited in different countries can have different implications, as seen in Jung et al. (2018). Their results suggested that students seemed less aware of things such as sex offender registries (SORs) and yet held more positive views of SORs than community members who were surveyed. Also, they found that those surveyed in the U.S. were more in favour of the availability of SORs than those surveyed in Canada. In a follow-up study with political decision-makers from Canada and the U.S., it became more apparent that those with conservative-leaning views perceived SORs as useful to protect the public (Jung et al., 2020). Hence, further research should examine whether person-first language makes a difference depending on the country. Our study and Snape and Fido’s study (2022) were conducted in Canada and the United Kingdom, respectively, whereas studies that found some differences, such as Harris and Socia’s (2016) and Lowe and Willis’ studies (2020), were conducted in the U.S. and New Zealand, respectively.

### Conclusion

In summary, person-first language did not lead to significant changes in negative perceptions when compared to other labels commonly used to describe persons who have sexually offended. We do not purport that the argument between the usage of the ‘sex offender’ label and person-first language is closed. The argument is framed in this study within the larger context of asking the question: does the label matter? The debate between premodified and postmodified nouns in the mental health literature suggests that it does matter (Granello & Gibbs, 2016). However, the body of empirical research on person-first language within a forensic context has a long way to go before any definitive conclusions can be made (Harris & Socia, 2016; Imhoff, 2015). In light of the literature demonstrating negative community perceptions may pose barriers to community reintegration, our study does not find that actively using person-first language directly changes public attitudes. However, adopting person-first language may be a step towards ensuring that individuals convicted of sexual offences are more likely to be treated with humanity and respect.

## Data Availability

The data that supports the findings of this study are available from the corresponding author upon reasonable request.

## Related Versions

This manuscript is derived from the second author’s undergraduate thesis completed at MacEwan University. The published article includes revised and expanded analyses that differ from those presented in the original thesis.

## Appendix

***Eight vignettes for this study’s 4 label conditions x 2 age of victim conditions*** 

1. Person-First Label Conditions 
  - Adult
    **Person convicted of a sexual offense to be released poses risk to reoffend, police warn**
    Police are warning the public about a person convicted of a sexual offense that poses a risk to offend again in the community. In the interest of public safety, ________ Police Service is issuing the following warning: ____ _____, 25, is set to be released from the ______ Penitentiary after serving two years for a conviction of sexual assault against an adult victim. He is considered by police to pose a risk of reoffending in the community. ______ will be residing in ________ but will be monitored closely by a specialized police unit. 
  - Child
    **Person convicted of a sexual offense to be released poses risk to reoffend, police warn**
    Police are warning the public about a person convicted of a sexual offense that poses a risk to offend again in the community. In the interest of public safety, ________ Police Service is issuing the following warning: ____ _____, 25, is set to be released from the ______ Penitentiary after serving two years for a conviction of sexual assault against a child victim. He is considered by police to pose a risk of reoffending in the community. ______ will be residing in ________ but will be monitored closely by a specialized police unit. 
2. ‘Sex offender’ Label Conditions 
  - Adult
    **Sex offender to be released poses risk to reoffend, police warn**
    Police are warning the public about a sex offender that poses a risk to offend again in the community. In the interest of public safety, ________ Police Service is issuing the following warning: ____ _____, 25, is set to be released from the ______ Penitentiary after serving two years for a conviction of sexual assault against an adult victim. He is considered by police to pose a risk of reoffending in the community. ______ will be residing in ________ but will be monitored closely by a specialized police unit. 
  - Child
    **Sex offender to be released poses risk to reoffend, police warn**
    Police are warning the public about a sex offender that poses a risk to offend again in the community. In the interest of public safety, ________ Police Service is issuing the following warning: ____ _____, 25, is set to be released from the ______ Penitentiary after serving two years for a conviction of sexual assault against a child victim. He is considered by police to pose a risk of reoffending in the community. ______ will be residing in ________ but will be monitored closely by a specialized police unit. 
3. Offense-Specific Label Conditions 
  - Adult
    **Rapist to be released poses risk to reoffend, police warn**
    Police are warning the public about a rapist that poses a risk to offend again in the community. In the interest of public safety, ________Police Service is issuing the following warning: ____ _____, 25, is set to be released from the ______ Penitentiary after serving two years for a conviction of sexual assault against an adult victim. He is considered by police to pose a risk of reoffending in the community. ______ will be residing in ________ but will be monitored closely by a specialized police unit. 
  - Child
    **Child molester to be released poses risk to reoffend, police warn**
    Police are warning the public about a child molester that poses a risk to offend again in the community. In the interest of public safety, ________ Police Service is issuing the following warning: ____ _____, 25, is set to be released from the ______ Penitentiary after serving two years for a conviction of sexual assault against a child victim. He is considered by police to pose a risk of reoffending in the community. ______ will be residing in ________ but will be monitored closely by a specialized police unit. 
4. Diagnostic Label Conditions 
  - Adult
    **Sexual sadist to be released poses risk to reoffend, police warn**
    Police are warning the public about a sexual sadist that poses a risk to offend again in the community. In the interest of public safety, ________ Police Service is issuing the following warning: ____ _____, 25, is set to be released from the ______ Penitentiary after serving two years for a conviction of sexual assault against an adult victim. He is considered by police to pose a risk of reoffending in the community. ______ will be residing in ________ but will be monitored closely by a specialized police unit. 
  - Child
    **Pedophile to be released poses risk to reoffend, police warn**
    Police are warning the public about a pedophile that poses a risk to offend again in the community. In the interest of public safety, ________ Police Service is issuing the following warning: _____ ____, 25, is set to be released from the ______ Penitentiary after serving two years for a conviction of sexual assault against a child victim. He is considered by police to pose a risk of reoffending in the community. ______ will be residing in ________ but will be monitored closely by a specialized police unit.

## References

[r1] American Psychological Association. (2020). *Publication manual of the American Psychological Association* (7th ed.).

[r2] Belisle, L. A., Marshall, E. A., & Butler, M. M. (2025). What about language? A mixed methods examination of the impact of person-centered language on students’ perceptions of individuals who commit sex offenses. Sexual Abuse: A Journal of Research and Treatment, 37(5), 503–528. 10.1177/10790632241283464 39262172

[r3] Bernburg, J. G. (2019). Labeling theory. In M. D. Krohn, N. Hendrix, G. Penly Hall, & A. J. Lizotte (Eds.), *Handbook of crime and deviance* (2nd ed., pp. 187-207). Springer Nature Switzerland.

[r4] Bernburg, J. G., Krohn, M. D., & Rivera, C. J. (2006). Official labeling, criminal embeddedness, and subsequent delinquency: A longitudinal test of labeling theory. Journal of Research in Crime and Delinquency, 43(1), 67–88. 10.1177/0022427805280068

[r5] Bogardus, E. S. (1925). Measuring social distance. Journal of Applied Sociology, 9, 299–308.

[r6] Bonta, J., & Andrews, D. A. (2024). *The psychology of criminal conduct* (7th ed.). Routledge.

[r7] Botha, M., Dibb, B., & Frost, D. M. (2022). “Autism is me”: An investigation of how autistic individuals make sense of autism and stigma. Disability & Society, 37(3), 427–453. 10.1080/09687599.2020.1822782

[r8] Calobrisi, E. A., & Knight, R. A. (2024). Comparing perceptions of individuals who sexually offend against children versus adults. Law and Human Behavior, 48(2), 133–147. 10.1037/lhb0000558 38602806

[r9] Cassidy, M., & Rydberg, J. (2020). Does sentence type and length matter? Interactions of age, race, ethnicity, and gender on jail and prison sentences. Criminal Justice and Behavior, 47(1), 61–79. 10.1177/0093854819874090

[r10] Church, W. T., II, Wakeman, E. E., Miller, S. L., Clements, C. B., & Sun, F. (2008). The Community Attitudes Toward Sex Offenders Scale: The development of a psychometric assessment instrument. Research on Social Work Practice, 18(3), 251–259. 10.1177/1049731507310193

[r11] Cochran, J. C., Toman, E. L., Shields, R. T., & Mears, D. P. (2021). A uniquely punitive turn? Sex offenders and the persistence of punitive sanctioning. Journal of Research in Crime and Delinquency, 58(1), 74–118. 10.1177/0022427820941172

[r12] Crocker, A. F., & Smith, S. N. (2019). Person-first language: Are we practicing what we preach? Journal of Multidisciplinary Healthcare, 12, 125–129. 10.2147/JMDH.S140067 30799931 PMC6371927

[r13] Feelgood, S., & Hoyer, J. (2008). Child molester or paedophile? Sociolegal versus psychopathological classification of sexual offenders against children. Journal of Sexual Aggression, 14(1), 33–43. 10.1080/13552600802133860

[r14] Ferguson, K., & Ireland, C. A. (2006). Attitudes towards sex offenders and the influence of offence type: A comparison of staff working in a forensic setting and students. British Journal of Forensic Practice, 8(2), 10–19. 10.1108/14636646200600009

[r15] Galeste, M. A., Fradella, H. F., & Vogel, B. (2012). Sex offender myths in print media: Separating fact from fiction in U.S. newspapers. Western Criminology Review, 13(2), 4–24.

[r16] Granello, D. H., & Gibbs, T. A. (2016). The power of language and labels: “The mentally ill” versus “people with mental illnesses”. Journal of Counseling and Development, 94(1), 31–40. 10.1002/jcad.12059

[r17] Hanson, R. K., Harris, A. J. R., Letourneau, E., Helmus, L. M., & Thornton, D. (2018). Reductions in risk based on time offense-free in the community: Once a sexual offender, not always a sexual offender. Psychology, Public Policy, and Law, 24(1), 48–63. 10.1037/law0000135

[r18] Hanson, R. K., Lee, S. C., & Thornton, D. (2024). Long term recidivism rates among individuals at high risk to sexually reoffend. Sexual Abuse: A Journal of Research and Treatment, 36(1), 3–32. 10.1177/10790632221139166 36382622 PMC11421192

[r19] Harris, A. J. R., & Hanson, R. K. (2004). *Sexual offender recidivism: A simple question* (Corrections User Report No 2004–01). Ottawa, Ontario, Canada: Public Safety and Emergency Preparedness Canada. https://www.publicsafety.gc.ca/cnt/rsrcs/pblctns/sx-ffndr-rcdvsm/index-en.aspx

[r20] Harris, A. J. R., & Socia, K. M. (2016). What’s in a name? Evaluating the effects of the “sex offender” label on public beliefs and opinions. Sexual Abuse: A Journal of Research and Treatment, 28(7), 660–678. 10.1177/1079063214564391 25542837

[r21] Helmus, L., Hanson, R. K., Thornton, D., Babchishin, K. M., & Harris, A. J. R. (2012). Absolute recidivism rates predicted by Static-99R and Static-2002R sex offender risk assessment tools vary across samples: A meta-analysis. Criminal Justice and Behavior, 39(9), 1148–1171. 10.1177/0093854812443648

[r22] Himmen, M. K., Thomas, M. L., Scavuzzo, R. R., & Jung, S. (2023). Can community notifications be improved to change public perceptions of released justice-involved persons and of the criminal justice system? International Journal of Law, Crime and Justice, 74, 100607. 10.1016/j.ijlcj.2023.100607

[r23] Hjeij, M., & Vilks, A. (2023). A brief history of heuristics: How did research on heuristics evolve? Humanities & Social Sciences Communications, 10, 64. 10.1057/s41599-023-01542-z

[r24] Hogue, T. E., Harper, C. A., & McAuliff, B. D. (2019). Development of a 21-Item short form of the Attitudes to Sexual Offenders (ATS) Scale. Law and Human Behavior, 43(1), 117–130. 10.1037/lhb0000308 30556704

[r25] Imhoff, R. (2015). Punitive attitudes against pedophiles or persons with sexual interest in children: Does the label matter? Archives of Sexual Behavior, 44(1), 35–44. 10.1007/s10508-014-0439-3 25501864

[r26] Jahnke, S., Blagden, N., & Hill, L. (2022). Pedophile, child lover, or minor-attracted person? Attitudes toward labels among people who are sexually attracted to children. Archives of Sexual Behavior, 51(8), 4125–4139. 10.1007/s10508-022-02331-6 36175817 PMC9663395

[r27] Jahnke, S., Imhoff, R., & Hoyer, J. (2015). Stigmatization of people with pedophilia: Two comparative surveys. Archives of Sexual Behavior, 44(1), 21–34. 10.1007/s10508-014-0312-4 24948422

[r28] Jensen, M. E., Pease, E. A., Lambert, K., Hickman, D. R., Robinson, O., McCoy, K. T., Barut, J. K., Musker, K. M., Olive, D., Noll, C., Ramirez, J., Cogliser, D., & King, J. K. (2013). Championing person-first language: A call to psychiatric mental health nurses. Journal of the American Psychiatric Nurses Association, 19(3), 146–151. 10.1177/1078390313489729 23698977

[r29] Jung, S. (2022). Applying RNR principles to effectively treat people who have committed a sexual offence. In K. Uzieblo, W. J. Smid, & K. McCartan (Eds.), *Challenges in the management of people convicted of a sexual offence* (pp. 157–172). Palgrave Macmillan.

[r30] Jung, S., Allison, M., & Martin, E. (2018). Perspectives of Americans and Canadians on the use and function of sex offender registries. International Journal of Law, Crime and Justice, 52, 106–117. 10.1016/j.ijlcj.2017.10.003

[r31] Jung, S., Allison, M., Toop, C., & Martin, E. (2020). Sex offender registries: Exploring the attitudes and knowledge of political decision-makers. Psychiatry, Psychology, and Law, 27(3), 478–492. 10.1080/13218719.2020.1733698 33071553 PMC7534266

[r32] Jung, S., Jamieson, L., Buro, K., & DeCesare, J. (2012). Attitudes and decisions about sexual offenders: A comparison of laypersons and professionals. Journal of Community & Applied Social Psychology, 22(3), 225–238. 10.1002/casp.1109

[r33] Katz-Schiavone, S., Levenson, J. S., & Ackerman, A. R. (2008). Myths and facts about sexual violence: Public perceptions and implications for prevention. The Journal of Criminal Justice and Popular Culture, 15(3), 291–311.

[r34] Kernsmith, P. D., Craun, S. W., & Foster, J. (2009). Public attitudes toward sexual offenders and sex offender registration. Journal of Child Sexual Abuse, 18(3), 290–301. 10.1080/10538710902901663 19856734

[r35] King, L. L., & Roberts, J. J. (2017). The complexity of public attitudes toward sex crimes. Victims & Offenders, 12(1), 71–89. 10.1080/15564886.2015.1005266

[r36] Kirsch, L. G., & Becker, J. V. (2007). Emotional deficits in psychopathy and sexual sadism: Implications for violent and sadistic behavior. Clinical Psychology Review, 27(8), 904–922. 10.1016/j.cpr.2007.01.011 17343965

[r37] Kruis, N. E., Ménard, K. S., Choi, J., Rowland, N. J., Frye, T., Kosaka, R., & Williams, A. (2025). Perceived dangerousness mediates punitive attitudes toward sex offenders: Results from a vignette experiment. Crime and Delinquency, 71(5), 1632–1662. 10.1177/00111287231170106

[r38] Levenson, J. S., Brannon, Y. N., Fortney, T., & Baker, J. (2007). Public perceptions about sex offenders and community protection policies. Analyses of Social Issues and Public Policy (ASAP), 7(1), 137–161. 10.1111/j.1530-2415.2007.00119.x

[r39] Levenson, J. S., Willis, G. M., & Prescott, D. S. (2020). Evidence-based practice and the role of trauma-informed care in sex offending treatment. In H. Swaby, B. Winder, R. Lievesley, K. Hocken, N. Blagden, & P. Banyard (Eds.), *Sexual crime and trauma* (pp. 197–224). Palgrave Macmillan.

[r40] Lowe, G., & Willis, G. (2020). “Sex offender” versus “person”: The influence of labels on willingness to volunteer with people who have sexually abused. Sexual Abuse: A Journal of Research and Treatment, 32(5), 591–613. 10.1177/1079063219841904 30957654

[r41] Lowe, G. T., & Willis, G. M. (2022). Do popular attitudinal scales perpetuate negative attitudes towards persons who have sexually offended? Journal of Sexual Aggression, 28(2), 231–243. 10.1080/13552600.2021.2009050

[r42] Magers, M., Jennings, W. G., Tewksbury, R., & Miller, J. M. (2009). An exploration of the sex offender specialization and violence nexus. Southwest Journal of Criminal Justice, 6(2), 133–144.

[r43] Malinen, S., Willis, G. M., & Johnston, L. (2014). Might informative media reporting of sexual offending influence community members’ attitudes towards sex offenders? Psychology, Crime & Law, 20(6), 535–552. 10.1080/1068316X.2013.793770

[r44] Martijn, F. M., Babchishin, K. M., Pullman, L. E., & Seto, M. C. (2020). Sexual attraction and falling in love in persons with pedohebephilia. Archives of Sexual Behavior, 49(4), 1305–1318. 10.1007/s10508-019-01579-9 32086644

[r45] Matsick, J. L., Kruk, M., Palmer, L., Layland, E. K., & Salomaa, A. C. (2022). Extending the social category label effect to stigmatized groups: Lesbian and gay people’s reactions to “homosexual” as a label. Journal of Social and Political Psychology, 10(1), 369–390. 10.5964/jspp.6823

[r46] McCoy, V. A., & DeCecco, P. G. (2011). Person-first language training needed in higher education. In *Ideas and research you can use: VISTAS 2011**.* https://manifold.counseling.org/projects/vistas-online-2011/resource/person-first-language-training-needed-in-higher-education

[r47] Mears, D. P., Mancini, C., Gertz, M., & Braton, J. (2008). Sex crimes, children, and pornography: Public views and public policy. Crime and Delinquency, 54(4), 532–559. 10.1177/0011128707308160

[r48] Olver, M. E., Wong, S. C. P., & Nickolaichuk, T. P. (2009). Outcome evaluation of a high-intensity inpatient sex offender treatment program. Journal of Interpersonal Violence, 24(3), 522–536. 10.1177/0886260508317196 18458350

[r49] Peer, E., Brandimarte, L., Samat, S., & Acquisti, A. (2017). Beyond the Turk: Alternative platforms for crowdsourcing behavioral research. Journal of Experimental Social Psychology, 70, 153–163. 10.1016/j.jesp.2017.01.006

[r50] Quinn, J. F., Forsyth, C. J., & Mullen-Quinn, C. (2004). Societal reaction to sex offenders: A review of the origins and results of the myths surrounding their crime and treatment amenability. Deviant Behavior, 25(3), 215–232. 10.1080/01639620490431147

[r51] Restivo, E., & Lanier, M. M. (2015). Measuring the contextual effects and mitigating factors of labeling theory. Justice Quarterly, 32(1), 116–141. 10.1080/07418825.2012.756115

[r52] Reynaert, C. C., & Gelman, S. A. (2007). The influences of language form and conventional wording on judgments of illness. Journal of Psycholinguistic Research, 36, 273–295. 10.1007/s10936-006-9045-4 17273932

[r53] Russell, S., Mammen, P., & Russell, P. S. S. (2005). Emerging trends in accepting the term *intellectual disability* in the world disability literature. Journal of Intellectual Disabilities, 9(3), 187–192. 10.1177/1744629505056693 16144824

[r54] Slovic, P., Finucane, M. L., Peters, E., & MacGregor, D. G. (2007). The affect heuristic. European Journal of Operational Research, 177(3), 1333–1352. 10.1016/j.ejor.2005.04.006

[r55] Snape, N., & Fido, D. (2022). Sex offenders vs. people with sexual offences: Putting the person before the offence. Journal of Concurrent Disorders, 4(3), 93–108. 10.54127/WASD7451

[r56] Socia, K. M., Rydberg, J., & Dum, C. P. (2021). Punitive attitudes toward individuals convicted of sex offenses: A vignette study. Justice Quarterly, 38(6), 1262–1289. 10.1080/07418825.2019.1683218

[r57] Spivey, E. D. (2024). “That doesn’t define who I am”: Strategies of resistance to stigmatization among a sample of U. S. individuals convicted of a sexual offense. Sexual Abuse: A Journal of Research and Treatment, 36(6), 714–744. 10.1177/10790632231200835 37670672

[r58] Tannenbaum, F. (1938). *Crime and the community*. Ginn and Company.

[r59] Tversky, A., & Kahneman, D. (1973). Availability: A heuristic for judging frequency and probability. Cognitive Psychology, 5(2), 207–232. 10.1016/0010-0285(73)90033-9

[r60] Tversky, A., & Kahneman, D. (1974). Judgment under uncertainty: Heuristics and biases. Science, 185(4157), 1124–1131. 10.1126/science.185.4157.1124 17835457

[r61] Weekes, J. R., Pelletier, G., & Beaudette, D. (1995). Correctional officers: How do they perceive sex offenders? International Journal of Offender Therapy and Comparative Criminology, 39(1), 55–61. 10.1177/0306624X9503900107

[r62] Wiley, S. A., & Esbensen, F.-A. (2016). The effect of police contact: Does official intervention result in deviance amplification? Crime and Delinquency, 62(3), 283–307. 10.1177/0011128713492496

[r63] Willis, G. M. (2018). Why call someone by what we don’t want them to be? The ethics of labeling in forensic/correctional psychology. Psychology, Crime & Law, 24(7), 727–743. 10.1080/1068316X.2017.1421640

[r64] Willis, G. M., Levenson, J. S., & Ward, T. (2010). Desistance and attitudes towards sex offenders: Facilitation or hindrance? Journal of Family Violence, 25(6), 545–556. 10.1007/s10896-010-9314-8

[r65] Wnuk, D., Chapman, J. E., & Jeglic, E. L. (2006). Development and refinement of a measure of attitudes toward sex offender treatment. Journal of Offender Rehabilitation, 43(3), 35–47. 10.1300/J076v43n03_03

[r66] Zack, E., Lang, J. T., & Dirks, D. (2018). “It must be great being a female pedophile!”: The nature of public perceptions about female teacher sex offenders. Crime, Media, Culture, 14(1), 61–79. 10.1177/1741659016674044

[r67] Zatkin, J., Sitney, M., & Kaufman, K. (2022). The relationship between policy, media, and perceptions of sexual offenders between 2007 and 2017: A review of the literature. Trauma, Violence & Abuse, 23(3), 953–968. 10.1177/1524838020985568 33438524

